# Knowledge, attitudes and practices of health professionals towards postoperative pain management at a referral hospital in Ethiopia

**DOI:** 10.1016/j.amsu.2021.103167

**Published:** 2021-12-14

**Authors:** Tadese Tamire Negash, Kumilachew Geta Belete, Wolderufael Tlilaye, Tamiru Tilahun Ayele, Keder Essa Oumer

**Affiliations:** a Department of Anesthesia, College of Health Sciences, Debre Tabor University, Debre Tabor, Ethiopia; b Department of Anesthesia, College of Health Sciences, Wolkite University, Wolkite, Ethiopia

**Keywords:** Pain, Post-operative pain, Postoperative pain management

## Abstract

**Background:**

*Postoperative* pain (POP) is a form of acute pain following surgery. It results from tissue injury during surgical procedure like skin incision, tissue dissection, manipulation and traction. It is one of the immediate postoperative complications. Despite new standards, guidelines and different strategies the practice of postoperative pain management is found to be inadequate. We aimed to assess knowledge, attitude and practice on postoperative pain management practice among Health professionals working at XX Referral Hospital.

**Method:**

*Institution* based cross-sectional study was conducted to assess Knowledge, Attitudes and Practices of Health professionals regarding to Post-operative pain management at XX Referral Hospital 2020 from 118 health professionals. Data was collected using structured self-administered questionnaire and was verified, coded and entered to Epi Info Software version 3.5.4 and then it was exported and analyzed by SPSS version 20 Software. After analysis frequency and percentages was used to summarize the finding.

**Result:**

The overall finding of the study revealed that health professionals had good knowledge (58.4%), unfavorable attitude (44.9%), and poor practice (24.58%) towards post-operative pain management.

**Conclusion:**

Non physician anesthetists have good knowledge, attitude and practice towards post-operative pain management. But the overall attitude and practice of health professionals’ towards post-operative pain management is poor.

## Highlights

• Attitude of HCPs towards post-operative pain management is unfavorable.
• Practice of post-operative pain management inadequate.
• Regular training and developing local protocols would help to fill the gap.

## 1 Introduction

**Fig. 1:**
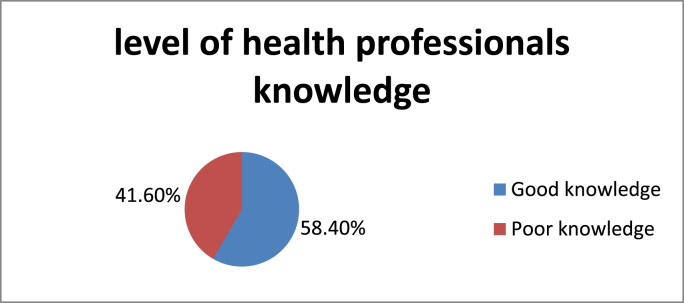
Health professionals' level of knowledge towards post-operative pain management in XX Referral Hospital, XX Ethiopia 2020.

**Fig. 2:**
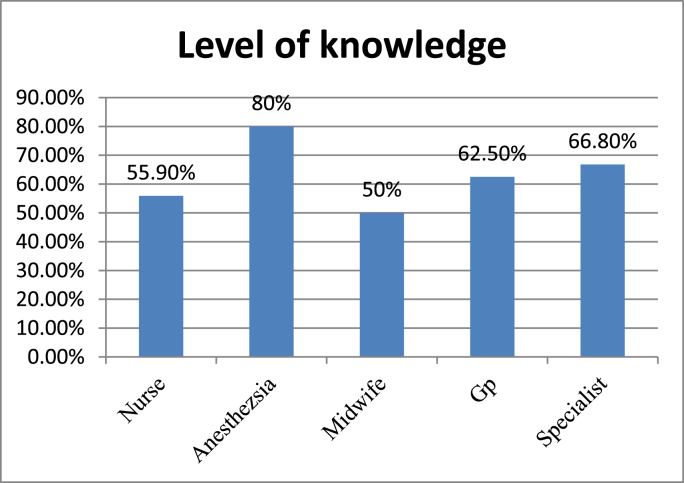
Level of knowledge each department towards post-operative pain management in XX Referral Hospital, XX Ethiopia 2020.

**Fig. 3:**
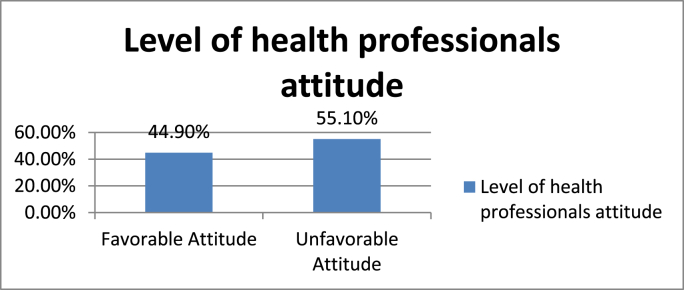
Health professionals' level of attitude towards post-operative pain management in XX Referral Hospital, XX Ethiopia 2020.

**Fig. 4:**
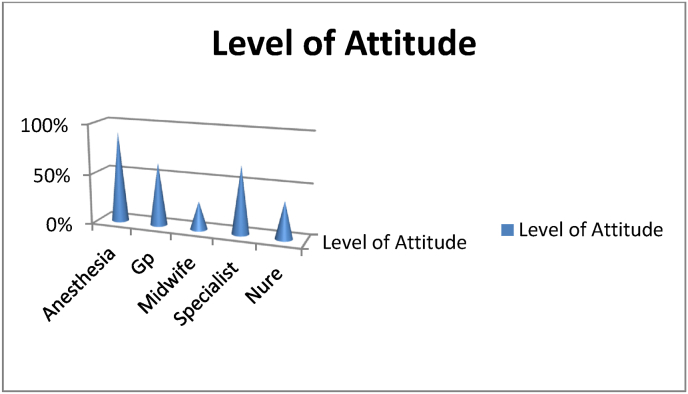
Each department's level of attitude towards post-operative pain management in XX Referral Hospital, XX Ethiopia 2020.

**Fig. 5:**
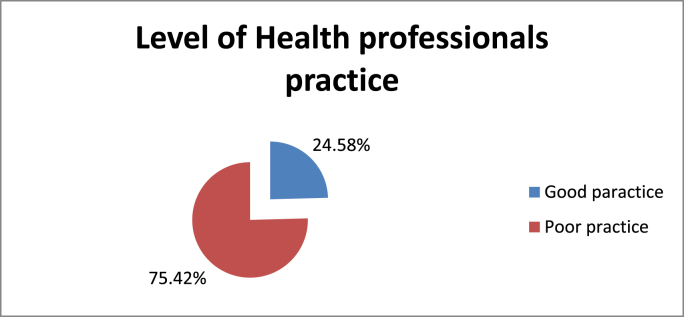
Health professionals' level of Practice towards post-operative pain management in XX Referral Hospital, XX Ethiopia 2020.

**Fig. 6:**
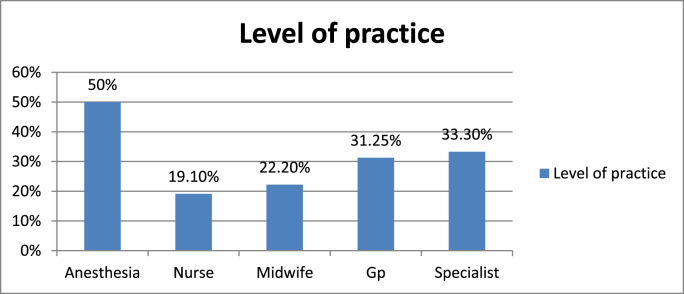
Level of practice of each department towards post-operative pain management in XX Referal Hospital, XX Ethiopia 2020.

The International Association for the Study of Pain defines pain as “an unpleasant sensory and emotional experience associated with actual or potential tissue damage, or described in terms of such damage.” [1].(see Table 1, Table 2, Table 3, Table 4, Fig. 1, Fig. 2, Fig. 3, Fig. 4, Fig. 5, Fig. 6)

**Table 1:** Socio-demographic characteristics of Health professionals at Debretabor referral hospital, Ethiopia, 2020.

Variables		Number (Percent %)
Marital status	Married	81(68.6%)
	Single	37(31.4%)
Gender	Female	40(33.9%)
	Male	78(66.1%)
Religion	Orthodox	107(90.7%)
	Muslim	8(6.8%)
	Protestant	3(2.5%)
Duration of service in year	<2 Year	10(8.5%)
	2–4 years	46(39%)
	5–9 years	39(33.1%)
	10–15 years	22(18.6%)
	>15 Year	1(0.8%)
Educational qualification	Nurse	68(57.6%)
	Midwife	19(16.1%)
	Anesthesia	10(8.5%)
	Gp	16(13.6%)
	Specialist	5(4.2%)
Level of education	Diploma	20(16.9%)
	Degree	69(58.5%)
	Masters	8(6.8%)
	Gp	16(13.6%)
	Other	5(4.2%)

**Table 2:** Frequency & percentage distribution of Health professions’ knowledge about POPM at XX referral hospital, Ethiopia, 2020.

Questions	Yes		No	
	Number	Percent	Number	Percent
The most accurate judge of the intensity of the patient's pain is the Patient	108	91.52.%	10	8.48%
Paracetamol injection is used in managing pop	105	89%	13	11%
Non pharmacological interventions are very effective for mild to moderate pain not severe pain	55	46.6%	63	53.4%
pharmacological methods: Opioid analgesic such as pethidine and morphine are used to relieve pain in POP	109	92.4%	9	7.6%
Giving narcotics on a regular schedule is preferred over ‘‘p.r.n.’’ schedule for continuous pain	92	78%	26	22%
performing nerve block used to relieve pop management	44	37.3%	74	62.7%
combining analgesics that work by different mechanisms may result in better pain control with fewer side effects than using a single analgesic agent	31	26.3%	87	73.7%
Pain should be assessed before and after administering pain drugs	98	83.1%	20	16.9%
Observation is part of the method used in pop assessment	118	100%	0	0%
Respiratory depression rarely occurs in patients who have been receiving stable doses of Opioid over a period of months	42	35.6%	76	64.4%
Distraction, for example, by the use of music or relaxation, can decrease the perception of pain	69	58.5%	49	41.5%

**Table 3:** Attitude of Health professionals towards POP management at XX referral hospital, Ethiopia, 2020.

Questions	Strongly agree	Agree	Disagree	Strongly Disagree
Pain is seen in the patient's behavior	74(62.7%)	34(28.8%)	10(8.5%)	0(0%)
Distraction reduces pain intensity	20(16.9%)	46(39%)	50(42.4%)	291.7%)
Nonpharmacological interventions are very effective for mild to moderate pain not severe pain	2(1.7%)	23(17.5%)	89(75.4%)	4(3.4%)
Surgical patients usually do experience pain more intense than medical patients	28(23.7%)	50(42.4%)	40(33.9%)	0(0%)
Health professionals experience affect POP management	12(10.2%)	97(82.2%)	9(7.6%)	0(0%)
Observable changes in vital sign must be relied on to verify patient's complain of severe pain	10(8.5%)	106(89.8%)	2(1.7%)	0(0%)
Performing nerve blocks for surgical patients is effective in reducing complication	10(8.5%)	36(30.5%)	70(59.3%)	2(1.7%)
Health professionals are best judges of the patient's pain intensity	28(23.7%)	50(42.4%)	25(21.2%)	15(12.7%)

**Table 4:** Practice of Health professionals towards POP management at XX referral hospital, Ethiopia, 2020.

Questions	Yes		No		Not applicable
	Number	Percent	Number	Percent	
I have active involvement in Postoperative pain management in my hospital.	88	74.6%	30	25.4%	0
Do you assess pain postoperatively?	16	13.6%	102	86.4%	
Do you prescribe opioid medications?	31	26.3%	67	56.8%	20(16.9%)
Are pain scores and management discussed with your staff?	27	22.9%	91	77.1%	0
Have you received training related to POP assessment and management?	87	73.7%	31	26.3%	0
Are she/he use a pain assessment tool	5	4.2%	113	95.8%	0
Do you have a pain guideline or standard in your organization?	15	12.7%	103	87.3%	
Do you perform nerve block for surgical pt. in a part of pop management	2	1.7%	13	11%	103(87.3%)
Do you write orders for postoperative pain after surgery	31	26.3%	69	58.5%	18(15.3)

Pain is a global health issue that requires the attention of the health community and its management is complex and multifactorial. It needs a deeper understanding of the barriers for proper and optimum pain management needs to be addressed in order to remedy the deficiencies among healthcare professionals and improve patient care. There are three barriers that have been reported regarding pain management practices, patients’ related barriers, and institution related barriers and healthcare professionals related barriers [[2], [3], [4], [5]].

Postoperative pain (POP) is a form of acute pain due to surgery. It results from tissue injury during surgical procedure like skin incision, tissue dissection, manipulation and traction [6].It is one of the most commonly seen therapeutic problems in patients admitted in hospital and it can increase morbidity(2). Various studies regarding prevalence of postoperative pain represent above 50% in first 24 h after surgery and above 30% in next 24 h after surgery [7].

Health professionals play a key role in pain assessment and in advising on the standards of pain management in postoperative recovery on surgical wards. They are the main providers of professional care within the postoperative care setting [8].

Failure to control postoperative pain may produce a range of detrimental acute and chronic effects. The attenuation of preoperative path physiology that occurs during surgery through reduction of nociceptive input to the central nervous system and optimization of preoperative analgesia may decrease complications and facilitate recovery during the immediate postoperative period and after discharge from the hospital [9,10].

Ineffective postoperative pain management may result in both direct and indirect costs. Direct costs include increased health care expenses due to increases in length of stay, use of medication, sick leave or residual disability. Indirect costs may include emotional upset, dissatisfaction, and financial burden due to disability. In view of these costs and the desire to reduce them, there is justification for the present study [11].

Knowledge deficiency regarding post-operative pain management is not the only factor that affects patients' well-being. Health professionals’ perceptions and satisfaction regarding pain management toward post-surgery patients also affect the quality of care [12].Their knowledge and ability to communicate are very important in pain management. Gap in knowledge about pain assessment and management, inability to assess pain, fear of side effects of analgesic drugs, inadequate staffing and poor communication between the patient and the health-care provider are all factors that lead to ineffective pain management [13,14].

There are still inadequacies of knowledge and attitude regarding post-operative pain management practice despite countless training courses, management guidelines, application strategies and multidisciplinary pain teams [15,16].

Due to inadequate availability of evidences to understand the knowledge, attitude and practice gap in the study area. It is inevitable to understand the knowledge, attitude and practice towards post-operative pain management status. Therefor this study aimed to determine the current health professionals’ knowledge, attitudes and practice regarding pain management practice working at XX Referral Hospital.

## 2 Methods

### 2.1 Study setting, design, period, and population

A hospital based cross-sectional study was conducted at XX Hospital, in North-central Ethiopia from November 01 to December 1, 2020. The Hospital gives medical, surgical, pediatrics, gynecologic, and obstetrics services. All Health professionals working at XX Referral Hospital were the source population while selected health professionals who have been working during the study period were the study population. Health professionals who have been on sick leave, annual leave and unavailable for some other reasons during the study period were excluded. This study is reported in line with STROCCS checklist [17] and registered at www.researchregistry.com with Research Registry UIN: researchregistry7376.

Ethical approval was obtained from the ethics review committee in DTU. In addition, permission to conduct the research was obtained from the administrative office of the hospital. Before the data collection, verbal consent was obtained from each participant. The study participants were informed about the purpose of the study, why and how they were selected. Moreover, Health professionals were told that they were free to withdraw from the study at any time during the research. Any specific identification was not included in the data collection tool and this was assured by using code numbers to each data and by analyzing the data in aggregate.

### 2.2 Sample size determination

Sample size was determined by taking the following assumption take the proportion 87.3% study result of Arusi zonal hospital, confidence interval of 95% and margin of error of to be tolerated 0.05.The sample size to be taken for the study will be determined using the formula.

n = z^2^ (p) (1-p)/ d^2^

Whereas n = sample size

Z = confidence interval (1.96)
P = estimated prevalence (0.5)
d = margin of sampling error to be tolerated (0.05). To get the sample size with confidence interval of 95% and margin of error 5%.
n= (1.96)^2^0.0.873(1-0.873) = 170

0.05^2^

By applying a finite population correction formula, the final sample size was,

NF = n/ (1+n/N)

Whereas NF = the minimum sample size

n = sample size
N = Total number of health care professional

NF = n/ (1+n/N)

170/1 + 170/283) = 107

By adding 10% non-responding rate total sample size was 118

### 2.3 Sampling technique

The final study subject was chosen by a convenience sampling technique.

#### 2.3.1 Operational definitions

Knowledge: means the Health professionals’ perception and understanding of post-operative pain management based on experience.

This categorize as good knowledge and low knowledge

Good Knowledge: is the Knowledge Status of Health professional when they scored more than the mean.

Low knowledge: is the Knowledge Status of Health professional when they scored less than the mean.

Attitude: refer to the Health professionals’ behavior and way of acting towards effective pain management.

This is categorized as Favorable attitude and Unfavorable attitude.

Favorable attitude: is the category of Health professional when they scored more than the mean value.

Unfavorable attitude: is the category of Health professional when they scored less than the mean value.

Practice: Means the Health professional skill on post-operative pain management based on their experience.

Good practice: is the practice Status of Health professional when they scored more than the mean.

#### 2.3.2 Data collection

Self-administered structured questionnaire was used to collect the data from study participants. The tool was developed and adapted after intense review of literatures. It was prepared in English and the questionnaire was contains four parts which include nurses’ socio-demographic status, knowledge of POP assessment and management, attitude of POP assessment and management and practice of POP assessment and management.

Data was collected by third year and fourth year anesthesia student. Training was given for three days for data collectors by the principal investigator to make them familiar with the data collection tool. Principal investigator was assisted and coordinates the data collectors.

Data was collected from Health professional that was select from each ward. The principal investigator took the responsibility of coordinating the health professionals and discussed about the purpose of the study. Then based on their willingness to participate, questionnaire was distributed, and orientation was given on how to fill the questionnaire and clarification for any difficulties.

#### 2.3.3 Data quality assurance

The questionnaire was initially prepared in English and was translated in to Amharic language and again back translated to English by another expert to check for its consistencies. Pretesting of the questionnaire was done at other Referral hospital by taking 5% of the total participants prior to data collection. Moreover, during data collection, supervisors were check how the data collection process was going on. At the end of each data collection day, the principal investigator and supervisors also were checked the completeness of filled questionnaires. Every questionnaire was checked before data entry by principal investigator.

#### 2.3.4 Data entry and analysis

After the completion of the data collection process the required data was categorized and recorded. Then the collected data was processed and analyzed using statistical package (SPSS) version 20. After data collection each questionnaire was cheeked for completeness then coded and entered in to INTR OP INFO version 7. Finally the result was presented by different graphs, tables and description in statements.

## 3 Result

### 3.1 Socio-demographic characteristics of health professions

A total of 118 structured questionnaires were distributed to different health worker in the hospitals in DTGH and the response rate was 100%. From all the respondents, 78 (66.1%) and 30 (33.9%) were male and female, respectively. The mean ages of participants were 29.33 years ±4.41 SD. The majority of respondents 107(90.7%) were orthodox Christian.69 (58.5%) were bachelor degree holders; 46(39%) had 2–4 years of work experience.

### 3.2 Knowledge of health professionals to post-operative pain management

All participants 118(100%) knew that Observation is part of the method used in pop assessment and 108(91.52%) of the study subjects said that the most accurate judge of the intensity of the pain is the Patient while 87(73.7%) of participants said multimodal analgesia that work by different mechanisms is not better pain control with fewer side effects than using a single analgesic agent.

A total of 16 questions were asked & the mean score was 9.68 with SD = 2.76 and minimum & maximum values are 1 & 16 respectively. Study participants who scored less than the mean value was regarded as poor knowledge whereas participants who scored more than the mean value were regarded as good knowledge. From 118 participants, 69(58.4%) had good knowledge and 49(41.6%) had poor knowledge about post-operative Pain management.

From those who had good knowledge 8(80%) Anesthesia, 38(55.9%) Nurse, 9(50%) Midwife, 10(62.5%) Gp and 4(66.8%) specialists have a good knowledge

### 3.3 Attitude of health professionals towards POP management

Almost all of the respondents 108(91.5%) thought that Pain was seen in the patient's behavior. Among the respondents 109(92.4%) thought that Health professionals experience affect POP management. 72(61%) of the respondent thought that performing nerve blocks for surgical patients is not effective in reducing complication.

Health professionals were asked to score 9 questions on a five-point Likert scale related to postoperative pain management. The mean score for attitude was 3.48 with SD = 0.46. Respondents who scored more than the mean value were regarded as having favorable attitude whereas who scored less than the mean value were regarded as having an unfavorable attitude towards post-operative pain management. Among the 118 respondents, 53(44.9%) had favorable attitude whereas 65(55.1%) of participants had unfavorable attitude towards post-operative pain management.

Among favorable attitude 9(90%), 25(36.8%), 10(62.5%), 5(27.8%) and 4(66.8%) were anesthesia, Nurse, Medical doctor, Midwife and Specialist

### 3.4 Practice of health professionals towards pain management

The majority of the respondents 88(74.6%) had active involvement in Postoperative pain management in their hospital. Among the respondents 102(86.4%) were not assess post-operative pain. 103(87.3%) of the respondent had no used standard guideline to treat post-operative pain.

The mean score for Health professions’ practice of post-operative pain management was 3.87 with a standard deviation of 1.65. Study participants who scored less than the mean value were regarded as having poor practice, whereas participants who scored more than the mean value were regarded as having good practice. Therefore, from all participants, 29(24.58%) had good practice and that of 89(75.42%) had poor practice.

According to the above figure Anesthesia 5(50%), Nurse 13(19.1%), Midwife 4(22.2%), Gp 5(31.25%) and Specialist 2(33.3%) had good practice.

## 4 Discussion

Pain treatment following surgery remains a serious medical concern. Poorly managed postoperative pain can cause delays in discharge and recovery, as well as make it impossible for to take part in rehabilitation programs, resulting in poor outcomes [18]. Regardless of recent developments in improved understanding of pain mechanisms, physiology and pharmacology, Publication and development of evidence based guidelines, formation of acute pain services (APSs), as well as initiatives such as ‘pain as the fifth vital sign’ and presence of new medications and devices post-operative pain management remained a major global concern [16,19].

In this study, we found that 58.4% of health professionals have good knowledge level towards postoperative pain management which is consistent with a study conducted to assess Knowledge, attitudes and practices of nurses regarding to post-operative pain management at hospitals of Arsi zone (54.5%), Ethiopia (56.5%) and Ghana (59%) [[20], [21], [22]]. However the results of this study were lower than those of studies conducted in Gondar (66%), Uganda (75%), Saudi Arabia (87.5%), Bangladesh(66.7%) the United Kingdom (73.8%), and United States of America (74%) of the study participants showed a strong understanding of post-operative pain management [[23], [24], [25], [26]]. The possible explanation for this discrepancy would be because of difference in socioeconomic level, study site, sample size and other factors like variety of data collecting tools.

Among different group of study participants 80% of non-physician anesthetists have good knowledge followed by specialist doctors (66.8) and general practitioners (62.5) and mid wives have lowest knowledge level among participants. The same finding has been shown in the study conducted in Jordan, Korea and Italy [2,27,28].This inter disciplinary knowledge difference would be due to educational curriculums difference and exposure to patients with post-operative pain. However this study shows a higher proportion of Anesthetists’ knowledge than a study conducted in Addis Ababa which illustrated low knowledge level of anesthetists towards post-operative pain management and this variation could be due to sample size and educational level variation of participants [29].

Despite having good knowledge, only 44.9% health professional had favorable attitude towards postoperative pain management. Most of the respondents thought that Pain was seen in the patient's behavior, believes the working experience of health professionals affect POP management and changes in vital sign must be relied on to verify patients complain of severe pain. For post-operative patients Pharmacological and non-pharmacological postoperative pain management should be started as fast as possible to suppress the development of peripheral and central sensitization which would occur due to untreated pain [30]. But in this study, most of participants believe non pharmacological pain management is useful for only mild and moderate postoperative pain. Effectiveness of nerve blocks as an integral part of multimodal analgesia for post-operative management has been shown in different literatures, however the in contrary to this majority of study participants thought that performing nerve blocks for surgical patients is not effective in reducing complication [[31], [32], [33], [34]].

The severity of pain a patient suffers after surgery is related to the extent of tissue damage and the type of surgery performed [35]. In Ethiopia, 88.2% of patients experience moderate to severe postoperative pain [36]. This study revealed poor postoperative pain management. Only 24.58% study participates had good practice and the remaining 75.42% had poor. Even if 73% participants have received training related to POP assessment and treatment only 14% of them assess POP regularly and 87% our study participants didn't have a pain guideline or standard in their organization. Yosef et al. has found lack of pain assessment tool, lack of management protocol, guideline and poor documentation as a barrier for post-operative pain management [37]. This idea also supported by the study in Hong Kong which showed having a postoperative pain management program designed in an evidence-based procedure in specific manner can reduce postoperative pain and improve patient satisfaction [38].

## 5 Conclusion

Even though non physician anesthetists have good knowledge, attitude and practice towards postoperative pain management, overall health professionals’ attitude and practice is poor. Giving regular training to the staffs, developing local protocol and management guideline would help to fill the existing gap.

## Availability of data and materials

Data and materials will be shared upon reasonable request.

## Ethical approval

Ethical clearance was obtained from Debre Tabor University, college of health sciences ethical clearance committee

## Sources of funding

Nothing to declare

## Provenance and peer review

Not commissioned, externally peer-reviewed

## Author contribution

All authors equally contributed to the study concept or design, data collection, data analysis or interpretation, writing the paper.

## Registration of research studies

1. Name of the registry: http://www.researchregistry.com
2. Unique Identifying number or registration ID: researchregistry7376.
Hyperlink to your specific registration (must be publicly accessible and will be checked): https://www.researchregistry.com/browse-the-registry#home/

## Consent

Informed consent was taken from study participants after telling them the aim of the study, benefit, harm of participating in the study, and they have been told as they can withdraw from the study at any step if they feel so.

## Guarantor

Mr. Keder Essa Oumer

## Declaration of competing interest

Nothing to declare

## Abbreviation

APS: American Pain Society
ASA: American Society of Anesthesiologists
DTRH: XX Referral Hospital
DTU: XX university
ETB: Ethiopian Birr
Gp: General practitioner
KAP: Knowledge Attitude Practice
POP: Post-Operative Pain
POPM: Post-Operative Pain Management
SPSS: Statistical Package for the Social Science
WHO: World Health Organization
